# Optimization and evaluation of venturi tube reactors for non-thermal milk pasteurization using CFD method

**DOI:** 10.1371/journal.pone.0349354

**Published:** 2026-05-19

**Authors:** Kimia Taki, Bahram Hosseinzadeh Samani, Sajad Rostami, Zahra Izadi, Firozeh Nazari

**Affiliations:** 1 Department of Mechanical Engineering of Biosystem, Shahrekord University, Iran; 2 Food and Drug Affairs Iran University of Medical Sciences, Tehran, Iran; University of Palermo: Universita degli Studi di Palermo, ITALY

## Abstract

With growing demand for preserving food’s nutritional and sensory qualities, non-thermal pasteurization methods like hydrodynamic cavitation in Venturi tubes offer an effective alternative to heat-based techniques. This study optimizes a Venturi reactor for milk pasteurization using computational fluid dynamics (CFD) with the k-ε turbulence model and response surface method (RSM). By adjusting throat diameter (4.91 mm), length (13.15 mm), and convergence/divergence angles (19.2°/8.31°), the design maximized turbulent kinetic energy (TKE) (0.2438 m²/s² peak, 0.0305 m²/s² average) and minimized energy loss (viscous dissipation 2.69 W, pressure drop 3483 Pa). Experiments confirmed a 2.91 log-reduction in *E. coli*, with negligible impact on milk’s fat, protein, and lactose, ensuring safety and quality.

## 1. Introduction

Pasteurization is one of the most important stages in food preservation. This method is widely used in the food industry to reduce microbial load and extend the shelf life of food products. Pasteurization dates back to the late 19th century when Louis Pasteur introduced it to improve food safety and increase the shelf life of products. Initially introduced for milk, this process gained official recognition in Chicago in 1908 [1,2]. Since then, it has been widely employed in dairy industries and the processing of liquid foods such as juices. However, in general, one of the main challenges of this method and thermal methods is the adverse effects on food products’ sensory and nutritional quality. Heat-sensitive food products, such as milk, undergo undesirable changes during pasteurization, including alterations in taste, smell, color, and even degradation of nutrients like vitamins. These issues have prompted an increase in research on non-thermal pasteurization methods [3,4].

One of the novel and effective approaches developed in this context is the cavitation phenomenon. The application of cavitation as a non-thermal pasteurization method represents a significant innovation in the food industry. In this method, gas bubbles are formed by controlling the fluid flow conditions and reducing pressure below the vapor pressure of the liquid. When the pressure returns to normal, these bubbles collapse, releasing significant energy. This energy destroys the structures of microorganisms without the need for direct heating, thereby preserving the nutritional and sensory properties of food products [5–7].

Turbulent kinetic energy (TKE) is crucial in analyzing transitional phenomena such as cavitation, mixing, energy loss, heat transfer, and mass transfer in fluids. Generally, this parameter is used to describe the intensity of turbulence within the reactor. The TKE generated in a venturi tube significantly eliminates microorganisms through the cavitation phenomenon. Increased TKE within the tube leads to the formation and collapse of cavitation bubbles that generate high-velocity micro-jets capable of damaging the cell walls of microorganisms, thereby inactivating them [8,9]. Numerous studies using experimental data and numerical simulations have investigated the relationship between turbulence and cavitation efficiency. [10] demonstrated that increased TKE in fluid flows can enhance the rate of cavitation bubble formation and stability. Over the years, various studies have been conducted on optimizing the cavitation process and enhancing TKE using venturi tube reactors in the food industry. The study conducted by [6] showed that the venturi hydrodynamic cavitation reactor is a very promising method in municipal wastewater treatment due to its simple design and low cost. [11] used a hydrodynamic cavitation reactor to disinfect seawater. This study found that hydrodynamic cavitation could be effectively used for microbial disinfection of seawater.

This research aims to design, simulate, and optimize a venturi tube reactor to create suitable conditions for generating cavitation phenomena and increasing TKE in the pasteurization process. Through numerical simulations and statistical analyses, this study seeks to investigate the effects of the reactor’s geometric variables and fluid flow parameters on cavitation, TKE, and energy loss within the tube, as well as to assess the efficiency of this system in non-thermal pasteurization processes while preserving the quality and sensory properties of food products.

## 2. Materials and methods

### 2.1. Venturi tube reactor geometry

The geometry of the hydrodynamic reactor consists of a simple design with a converging section (throat) and a diverging section (diffuser). The mechanism of this tube operates according to Bernoulli’s principle, where the fluid velocity increases as it passes through the throat while its static pressure decreases. The pressure drop balances any increase in TKE that the fluid may gain from increased speed through a contraction. Additionally, in the diverging section of the venturi tube (the diffuser), the speed decreases, and the pressure increases.

**Fig 1:**
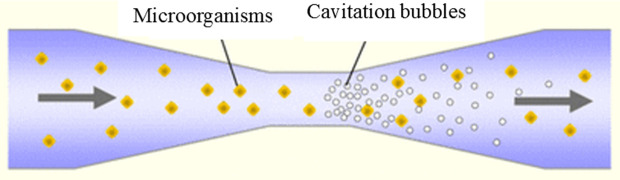
Hydrodynamic venturi tube cavitation reactor.

Hydrodynamic cavitation phenomena may also be generated by the pressure drop resulting from the change in fluid flow velocity within the reactor. In this case, the fluid pressure decreases and approaches the saturation vapor pressure, leading to local evaporation of the fluid. During this reaction, bubbles form at the throat of the reactor, and upon reaching high-pressure points (the diverging section of the reactor), explosions occur. These explosions release energy, promote mixing and agitation, and disrupt the colonies of microorganisms within the liquid [12,13]. Fig 1 shows a hydrodynamic venturi tube cavitation reactor.

**Fig 2:**
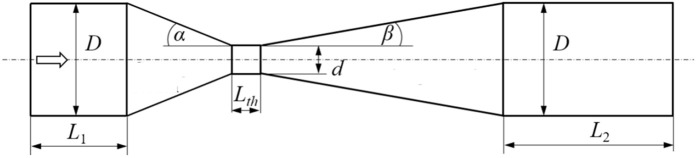
Structural parameters of the venturi tube.

Important structural parameters in the design and construction of a venturi tube include the tube diameter (D), throat diameter (d), convergence angle (α), throat length (Lth), and divergence angle (β). In this research, the inlet and outlet diameters of the tube are kept constant at 20 mm. Additionally, the tube material is considered to be glass. The overall geometry of the tube and its important parameters are illustrated in the accompanying figures. Fig 2 shows that the parameters L_1_ and L_2_ lengths are also fixed at 60 mm.

One of the most critical parameters in designing this reactor is obtaining the Reynolds number of the flow in the throat of the venturi tube (Equation 1).


$$\begin{array}{c} Re=\frac{\rho Ud}{\mu } \end{array}$$ (1)

Where 𝛒 is the fluid density (kg/m^3^), U is the fluid velocity in the pipe throat (m/s), D is the diameter of the throat (m), and 𝜇 is the viscosity of the desired fluid (pa.s). It also shows this type of flow inside the pipe.

laminar flow: Re < 2000

Transient current: 2000 < Re < 4000

Turbulent flow: Re > 4000

In this study, the obtained Reynolds number should be related to the range of turbulent flow.

In order to design this reactor, the diameter of the pipe throat can be calculated from Equation (2)


$$\begin{array}{c} \text{d }=(\text{4}*\text{Q})/((\pi *\beta *\Delta P) \end{array}$$ (2)

In this regard, Q is the transfer rate of the fluid flow inside the venturi tube, β is the discharge coefficient (from 0.6 to 0.99), and 𝛥𝑃 is the pressure drop. Also, by using Equations (2) and (3), the actual and theoretical current transfer rates can be calculated.


$$\begin{array}{c} \text{Q }=\beta *{\text{Q}}_{\text{Th}} \end{array}$$ (3)

$$\begin{array}{c} {\text{Q}}_{\text{Th}}={\text{A}}_{\text{Th}}*{\text{V}}_{\text{Th}} \end{array}$$ (4)

In these equations, Q is the actual flow rate, Q_Th_ is the theoretical flow rate, A_Th_ is the surface area of the venturi throat, and V_Th_ is the velocity in the venturi throat. Also, the velocity of the fluid in the throat of the venturi tube can be obtained through Equation (5).


$$\begin{array}{c} {\text{V}}_{\text{th}}=(\text{2}*\Delta \text{p}/\rho ) \end{array}$$ (5)

In this equation, 𝛒 is the fluid density. Based on the above equations, the ranges obtained for the reactor design in the current research are summarized in the accompanying Table 1.

**Table 1:** Obtained range of reactor dimensions.

L_th_	5-20 mm
D	3-16 mm
α	15˚-20˚
β	5˚-15˚

The flow regime was characterized using the Reynolds number (Equation 1). For the inlet (diameter D = 20 mm, velocity U = 0.3 m/s, density ρ = 1030 kg/m³, viscosity μ = 0.002 Pa·s), Re ≈ 3090, indicating transitional flow. At the throat, Re varies with diameter (d = 3–16 mm). For the optimized throat (d = 4.91 mm), the velocity is ~ 4.98 m/s (via mass continuity: U_throat = U· (D/d)²), yielding Re ≈ 12,590, confirming turbulent flow. For the largest throat (d = 16 mm), Re ≈ 2318, indicating transitional flow. These calculations confirm the suitability of the k-ε turbulence model for turbulent-dominated regimes in smaller throats, where cavitation is most pronounced.

### 2.2. Computational fluid dynamics (CFD) system simulation

In this part of the research, single-phase simulation was conducted using the CFD methods in software. The simulation was performed in stationary mode. Fluid flow was simulated under turbulence conditions using the k-ε turbulence model. The k-ε turbulence model, the most widely used model in CFD, is employed to simulate turbulent flow characteristics. The k-ε model was selected for its efficiency and reliability in simulating intense turbulent flows, ideal for analyzing TKE and its dissipation rate in the venturi reactor for non-thermal pasteurization. It provides an effective balance between accuracy and computational simplicity, unlike models such as k-ω SST, which are better suited for near-wall precision, making it appropriate for focusing on bulk flow dynamics in this study.

In this study, a 2-D, single-phase, steady-state Reynolds-Averaged Navier-Stokes (RANS) model with the standard k-ε turbulence closure was employed to predict regions of elevated TKE and high shear, serving as proxies for hydrodynamic cavitation-prone zones. This simplified approach, chosen for computational efficiency, does not directly resolve multiphase cavitation dynamics (bubble nucleation and collapse), with cavitation effects inferred from TKE levels. Due to the preliminary nature of the simulations, a formal grid convergence index (GCI) study and y⁺ analysis were not initially conducted. However, based on typical mesh configurations for Venturi tube simulations and flow conditions (throat diameter 4.91 mm, throat velocity ~4.98 m/s, milk properties: ρ = 1030 kg/m³, μ = 0.002 Pa·s), we estimate y⁺ ≈ 3–5 for a near-wall mesh spacing of 20–50 μm, suitable for the k-ε model with standard wall functions. A preliminary GCI analysis, assuming three structured meshes (coarse: ~ 50,000 cells; medium: ~ 150,000 cells; fine: ~ 450,000 cells) with a refinement ratio of ~1.73, suggests GCI < 2% for TKE max, indicating numerical reliability. These estimates ensure adequate mesh resolution for the study’s objectives. Limitations include the inability to directly simulate transient or multiphase cavitation phenomena; future studies will incorporate comprehensive GCI and y⁺ analyses and may employ cavitation-specific models (Schnerr-Sauer) for enhanced accuracy.

Additionally, the mesh configuration was chosen as normal for this simulation. Equations related to velocity, pressure, TKE, and turbulent energy dissipation (TED) rate were solved in 2-D domain conditions. Four key input variables for the simulation include the throat length and diameter (in millimeters) and the divergent and convergence angles of the venturi tube (in degrees). These input parameters were used to investigate their effect on the output variables, which include TKE in the area, TED rate, pressure drop, and total viscous dissipation (VD).

#### 2.2.1. K-ε turbulence model.

The governing fluid flow equations in this research are the Navier-Stokes equations, which have been solved using Reynolds-averaged Navier-Stokes (RANS). As mentioned earlier, CFD was used to perform this simulation. The K-epsilon (k-ε) turbulence model, the most widely used model in CFD, is employed to simulate turbulent flow characteristics. This two-equation model separately calculates the TKE and TED rate (ε). This model is particularly suitable for simulating turbulent flows with significant variations [14,15] (Equation 6, 7 and 8).


$$\begin{array}{c} \mu_{t}=\rho C_{\mu }\frac{k^{\text{2}}}{\text{€}} \end{array}$$ (6)

$$\begin{array}{c} \frac{\partial }{\partial \text{t}}(\rho \text{k})+\frac{\partial }{\partial \text{x}\text{i}}(\rho \text{ku}\text{i})=\frac{\partial }{\partial \text{x}\text{j}}((\mu +\frac{\mu_{\text{t}}}{\sigma_{\text{k}}})\frac{\partial_{\text{k}}}{\partial_{{\text{x}}_{\text{j}}}})+{\text{G}}_{\text{k}}+{\text{G}}_{\text{b}}-\rho_{\text{€}}-{\text{Y}}_{\text{M}}+{\text{S}}_{\text{k}} \end{array}$$ (7)

$$\begin{array}{c} \frac{\partial }{\partial \text{t }}\left(\rho_{\text{€}}\right)+\frac{\partial }{\partial \text{x}\text{i}}\left(\rho_{\text{€}ui}\right)=\frac{\partial }{\partial \text{xj}}(\left(\mu +\frac{\mu_{t}}{\sigma_{\text{€}}}\right))\frac{\partial }{\partial \text{x}\text{j}}+C_{\text{1}\text{€}}\frac{\text{€}}{k}\left(G_{k}+G_{\text{3}\text{€}}G_{b}\right)-C_{\text{2}\text{€}}\rho \frac{{\text{€}}^{\text{2}}}{K}+S_{\text{€}} \end{array}$$ (8)

In these equations, the constants of the model are:


$$\begin{array}{c} C_{\text{1}\text{€}}=\text{1}.\text{4}\text{4}C_{\text{2}\text{€}}=\text{1}.\text{9}\text{2} \end{array}$$

$$\begin{array}{c} C_{\mu }=\text{0}.\text{0}\text{9}\sigma_{K}=\text{1}.\text{0} \end{array}$$

$$\begin{array}{c} \sigma_{\text{€}}=\text{1}.\text{3} \end{array}$$

$G_{k}$: energy production due to the gradients of the average flow velocity.

$G_{b}$: Energy production is due to buoyancy (it is zero for flows without gravity and heat transfer).

$Y_{M}:$ Kinetic energy production due to flow compressibility effects (it is zero for incompressible flows).

$\sigma_{\varepsilon }{,\sigma }_{k}\;:$ Parantel numbers corresponding to k and ɛ.

$S_{\varepsilon },S_{k}:$ source terms definable by the operator.

#### 2.2.2. TKE.

The TKE is a physical quantity that describes the amount of energy in the velocity fluctuations caused by the turbulence of a fluid flow, and it is one of the most important parameters of this research because the higher the TKE, the better the mass transfer mixing, and agitation within the venturi tube, and also, the probability of cavitation phenomenon increases [16]. This parameter is obtained from the Equation (9).


$$\begin{array}{c} TKE=\frac{\text{1}}{\text{2}}({\overset{―}{\upsilon }}^{\text{2}}+{\overset{―}{\nu }}^{\text{2}}+{\overset{―}{\omega }}^{\text{2}}) \end{array}$$ (9)

In this equation, u, v, and w are the components of velocity fluctuations in x, y, and z directions, and (u ¯’)2, (v ¯’)2, and (w ¯’)^2^ are the mean squares of the velocity fluctuations in three coordinate directions.

#### 2.2.3. TED rate.

The dissipation of kinetic energy refers to the rate at which the kinetic energy generated by turbulence is converted into thermal energy (heat) due to viscous forces within a fluid. The TED rate is obtained from the velocity gradients based on the following Equation (10).


$$\begin{array}{c} \varepsilon =\frac{\text{1}}{\text{2}}\text{ʋ}{\left(\frac{\overset{―}{\partial Ut}}{\partial xJ}+\frac{\overset{―}{JU\partial }}{Lx\partial }\right)}^{\text{2}} \end{array}$$ (10)

Where ε is the TED rate (m^2^/s^3^), ʋ is the kinematic viscosity of the fluid (m^2^/s), u_i_ is the velocity component in the i-th direction and x_j_ spatial coordinate in the j-th direction [17].

#### 2.2.4. Pressure drop (*𝛥*P).

The pressure drop within the venturi tube is explained by Bernoulli’s principle. Initially, the fluid passes through a wide section and then reaches a narrow section, which is the throat of the tube. When the fluid enters the throat, the cross-section decreases, causing the fluid velocity to increase. According to Bernoulli’s principle, a pressure drop occurs as the fluid velocity increases. Therefore, the fluid pressure reaches its lowest point at the tube’s throat. As the fluid continues to flow past the throat and the cross-sectional area increases to its original size, the pressure increases again. pressure drop can be used to determine the system’s efficiency. The pressure drop is given by the Equation (11).


$$\begin{array}{c} \Delta \text{P }={\text{P}}_{\text{2}}-{\text{P}}_{\text{1}} \end{array}$$ (11)

P_1_ is the minimum reactor pressure, and P_2_ is the maximum reactor pressure.

#### 2.2.5. VD.

VD refers to converting kinetic energy into thermal energy due to internal friction within a fluid, which affects the stability and flow characteristics. This dissipation is calculated using the following Equation (12).


$$\begin{array}{c} \Phi =\text{2}\mu {\left(\frac{\partial u}{\partial x}\right)}^{\text{2}}+\text{2}\mu {\left(\frac{\partial v}{\partial y}\right)}^{\text{2}}+\text{2}\mu {\left(\frac{\partial w}{\partial z}\right)}^{\text{2}} \end{array}$$ (12)

Where μ is the dynamic viscosity of the fluid, u, v, and w are the components of the fluid velocity in three coordinate directions, ∂u/∂x, ∂v/∂y, and ∂w/∂z are the velocity gradients in the three coordinate directions that determine the rate of velocity changes they show [18,19].

#### 2.2.6. Fluid.

In this simulation, the reactor’s contents are considered whole milk with a fat content of 1.4% and 37% solids. Since milk is a very important food consumed from the beginning of life until middle age, requiring sanitization and purification, it has been chosen as the fluid under investigation in this study for pasteurization and microorganism elimination. The viscosity of the milk is assumed to be 0.002 Pa·s, and its density is considered to be 1030 kg/m³.

#### 2.2.7. Boundary conditions.

In this simulation, the inlet fluid velocity is set to 0.3 m/s, and the outlet reactor pressure is 0 Pa. Additionally, the walls are assumed to be no-slip boundaries.

Boundary conditions were defined as a fixed inlet velocity of 0.3 m/s was applied, consistent across all simulations. Gauge pressure was used, with the outlet set to 0 Pa. Absolute pressures were estimated relative to atmospheric pressure (101,325 Pa). At the throat, the pressure drops below the milk vapor pressure (~2300 Pa at 20°C), indicating potential for hydrodynamic cavitation.

Ultimately, based on all the details provided regarding the simulation procedure and numerical setup, the key parameters and conditions of the CFD analysis are summarized in Table 2.

**Table 2:** Summary of the CFD setup and simulation parameters.

Parameter	Value
Simulation type	2-D, single-phase, steady-state RANS
Turbulence model	Standard k–ε
Mesh type	Normal
Fluid	Whole milk (1.4% fat, 37% solids)
Density	1030 kg/m
Dynamic viscosity	0.002 Pa·s
Inlet condition	Velocity inlet, 0.3 m/s
Outlet condition	Pressure outlet, 0 Pa
Wall condition	No-slip
Output variables	TKE, TED rate, pressure drop, viscous dissipation (VD)

### 2.3. Modeling and optimization of simulation parameters

In this study, the RSM was used to optimize the geometric dimensions of the reactor and examine the effect of various geometric variables on the simulation output parameters. This method models the nonlinear relationships between input and output variables and performs optimization. By considering the defined upper and lower limits for each variable and the number of variables, RSM generates an experimental matrix, determining the number of tests and the levels of each variable for each test. This method is particularly suitable when the number of variables is high. Additionally, it determines the optimal values by solving the Equation (13).


$$\begin{array}{c} {\text{Y}}_{\text{i}}=\beta_{\text{0}}+\Sigma \beta_{\text{i}}{\text{X}}_{\text{i}}+\Sigma \beta_{\text{ij}}{\text{X}}_{\text{i}}{\text{X}}_{\text{j}}+\Sigma \beta_{\text{jj}}\overset{\text{2}}{\underset{\text{1}}{\text{X}}}+\varepsilon \end{array}$$ (13)

Where β_0_, β_i_, β_ij_, and βjj are constant coefficients, X_i_ and X_j_ are the independent process variables, and ɛ is the random error. The Box-Behnken design was used in this experiment, and the selected levels for the independent variables were chosen according to Table 3.

**Table 3:** Selected independent variables on response surface method.

Independent variable	Codded level		
	1	0	−1
L_th_ (mm)	5	12.50	20
α (degree)	15	17.50	20
β (degree)	5	10	15
d_th_ (mm)	3	9.50	15

The Box-Behnken design comprised 26 experiments and 3 replicates, and analysis of variance (ANOVA) was performed to assess the statistical significance of input variables (throat length, throat diameter, convergence/divergence angles) to optimize TKE and minimize energy dissipation for non-thermal pasteurization.

Furthermore the effects of the independent parameters on the throat length (mm), convergence angle (degree), divergence angle (degree), and throat diameter (mm) were analyzed [20].

### 2.4. Preparation of samples and microbial tests

To explore the correlation between TKE and *E. coli* inactivation in milk, fresh milk was used as the experimental medium. The milk samples were sterilized using autoclaves to eliminate pre-existing microorganisms, ensuring a controlled environment with only inoculated *E. coli*. Fresh milk samples from Shahrekord University’s dairy farm were autoclaved (121°C, 15 minutes) to ensure sterility. The milk was then inoculated with an *E. coli* suspension to achieve an initial concentration of ~10^4 colony-forming units per milliliter (CFU/mL). Control samples (inoculated milk without processing) and treated samples processed through the venturi reactor were cultured on MacConkey Agar (250 μL per dilution, three dilutions per sample) and incubated at 37°C for 24 hours. Bacterial reduction was assessed using the standard plate count method. The samples were then processed using the venturi tube reactor under different operational conditions designed to vary TKE levels, as determined by CFD simulations. Each milk sample was recirculated through venturi reactors of varying dimensions for 10 minutes to ensure consistent exposure to cavitation effects across different reactor configurations.

Microbial counts were measured before and after treatment using the standard plate count method. Viable cell counts were determined by plating treated samples on selective media, followed by incubation at 35 °C for 24 hours. The resulting colony-forming units (CFUs) were recorded. The experimental data were analyzed to identify the correlation between TKE levels and *E. coli* reduction. This analysis provided critical insights into optimizing reactor design and operational parameters to enhance microbial inactivation efficiency in milk [21–23].

Experiments were conducted using a centrifugal pump (0.5–1 bar inlet pressure, controlled via a PID valve to maintain a constant inlet velocity of 0.3 m/s). The Venturi tube was made of borosilicate glass (surface roughness < 10% for CFU counts).

### 2.5. Evaluation of qualitative properties

In this study, experiments were conducted with the aim of examining and evaluating the effect of the combined system on the quality properties of milk, including fat, protein, lactose, SnF (Solid-not-Fat), FFp (Fat-Free portion) and pH.

A digital pH meter was used to measure pH. For this measurement, the milk was left at room temperature for a while, the pH meter was calibrated, and after cleaning its electrode with distilled water, it was placed into the milk and then the number on the pH meter was noted.

Also, a milk scanner was used to determine the amounts of lactose, fat, SnF, FFp and protein in milk. The operation of this device was very simple and it measured several quality parameters of milk at the same time. For this purpose, after turning on the device, the milk sample was placed in a special container of the device and its special probe was placed inside the milk sample and finally the measured parameters of the milk were displayed on the device’s screen.

## 3. Results and discussion

In this study, the design of a hydrodynamic venturi tube reactor focused on four geometric parameters: throat length (mm), convergence angle (degree), divergence angle (degree), and throat diameter (mm). Based on a review of previous sources and the previously mentioned equations, a specific range was defined for each parameter, including throat length from 5 mm to 20 mm, convergence angle from 15 ˚ to 20 ˚, divergence angle from 5 ˚ to 15 ˚, and throat diameter ranging from 3 mm to 16 mm [23]. Subsequently, Design Expert software and the response suface method (RSM) were employed to optimize the reactor dimensions. Based on the dimensions obtained from this software, fluid flow simulations within the reactor were conducted using CFD methods [21].

### 3.1. Analysis method and test optimization

The hydrodynamic venturi tube reactor’s dimensions were optimized using Design Expert software and the RSM. In this study, the throat length (mm), convergence angle (degree), divergence angle (degree), and throat diameter (mm) were considered independent experimental variables. At the same time, the maximum TKE (TKE max) and average TKE (TKE ave), TED rate, 𝛥P, and VD were the dependent experimental variables. Based on the conducted analysis, two equations (actual and coded equation) were extracted for each dependent variable in this experiment. The Equations (14–18) and (19–23) represent the actual equation for the dependent experimental variables, which correspond to the maximum TKE, TKE ave, TED rate, 𝛥P, and VD, respectively.


$$\begin{array}{cc} {\text{TKE }}_{\text{max}}= & +\text{0}.\text{98041 }-\text{0}.\text{16117 }*\text{ Alpha }+\text{0}.\text{070753 }*{\text{d}}_{\text{th}}-\text{6}.\text{55333E}-\text{003 }*\text{ Alpha }*{\text{d}}_{\text{th}}\\ & +\text{1}.\text{70933E}-\text{003 }*{\text{d}}_{{\text{th}}^{\text{2}}} \end{array}$$ (14)

$$\begin{array}{cc} {\text{TKE }}_{\text{ave}}=-\text{0}.\text{096715 }+\text{8}.\text{25185E}-\text{003 }*\text{ Alpha }+\text{4}.\text{57895E}-\text{003 }*{\text{d}}_{\text{th}}-\text{7}.\text{00019E}-\text{004 }*\\ & \text{Alpha }*{\text{d}}_{\text{th}}+\text{2}.\text{95733E }-\text{ 004 }*{\text{d}}_{{\text{th}}^{\text{2}}} \end{array}$$ (15)

$$\begin{array}{cc} \text{TED}=-\text{1}.\text{93970 }+\text{0}.\text{15024 }*\text{ Alpha }+\text{0}.\text{079031 }*\text{ Beta }+\text{0}.\text{044769 }*{\text{d}}_{\text{th}}-\text{0}.\text{012647 }*\\ & \text{Alpha }*{\text{d}}_{\text{th}}-\text{6}.\text{60764E}-\text{003 }*\text{ Beta }*{\text{d}}_{\text{th}}+\text{9}.\text{40529E}-\text{003 }*{\text{d}}_{{\text{th}}^{\text{2}}} \end{array}$$ (16)

$$\begin{array}{c} \Delta \text{p}=-\text{8379}.\text{61846 }+\text{782}.\text{39248 }*\text{ Alpha }+\text{320}.\text{45716 }*{\text{d}}_{\text{th}}-\text{65}.\text{53023 }*\text{ Alpha }*{\text{d}}_{\text{th}}+\text{31}.\text{33887 }*{\text{d}}_{{\text{th}}^{\text{2}}} \end{array}$$ (17)

$$\begin{array}{cc} \text{VD}=-\text{6}.\text{05134 }+\text{0}.\text{52030 }*\text{ Alpha }+\text{0}.\text{26335 }*\text{ Beta }+\text{0}.\text{067538 }*{\text{d}}_{\text{th}}-\text{0}.\text{044468 }*\\ & \text{Alpha }*{\text{d}}_{\text{th}}-\text{0}.\text{022297 }*\text{ Beta }*{\text{d}}_{\text{th}}+\text{0}.\text{036239 }*{\text{d}}_{{\text{th}}^{\text{2}}} \end{array}$$ (18)

By removing the insignificant coefficients and making the remaining coefficients dimensionless, the coded equations of each dependent variable were obtained from the actual equation. Equations (19–23) show the coded equation of TKE max, TKE ave, TED rate, 𝛥p, and VD.


$$\begin{array}{c} {\text{TKE }}_{\text{max}}=-\text{3}.\text{022E}-\text{003 }+\text{0}.\text{041}*\text{ B }-\text{0}.\text{074 }*\text{ D }-\text{0}.\text{11}*\text{ B }*\text{ D }+\text{0}.\text{043 }{\text{B}}^{\text{2}}*+\text{0}.\text{072 }{\text{D}}^{\text{2}} \end{array}$$ (19)

$$\begin{array}{c} {\text{TKE }}_{\text{ave}}=+\text{1}.\text{504E}-\text{003 }+\text{4}.\text{004E}-\text{003 }*\text{ B }-\text{0}.\text{013 }*\text{ D }-\text{0}.\text{011}*\text{ B }*\text{ D }+\text{0}.\text{012}*{\text{D}}^{\text{2}} \end{array}$$ (20)

$$\begin{array}{c} \text{TED}=+\text{0}.\text{024 }+\text{0}.\text{075 }*\text{ B }+\text{0}.\text{081 }*\text{ C }-\text{0}.\text{42}*\text{ D }-\text{0}.\text{21}*\text{ B }*\text{ D }-\text{0}.\text{21}*\text{ C }*\text{ D }+\text{0}.\text{40 }*{\text{D}}^{\text{2}} \end{array}$$ (21)

$$\begin{array}{c} \Delta \text{p}=+\text{290}.\text{53 }+\text{ 399}.\text{64}*\text{ B }-\text{ 1500}.\text{74}*\text{ D }-\text{ 1064}.\text{87}*\text{ B }*\text{ D }+\text{ 1324}.\text{07}*{\text{D}}^{\text{2}} \end{array}$$ (22)

$$\begin{array}{c} \text{VD}=+\text{0}.\text{089}++\text{0}.\text{24}*\text{ B }+\text{0}.\text{26 }*\text{ C }-\text{1}.\text{59 }*\text{ D }-\text{0}.\text{72}*\text{ B }*\text{ D }-\text{0}.\text{72}*\text{ C }*\text{ D }+\text{1}.\text{53}*{\text{D}}^{\text{2}} \end{array}$$ (23)

In these equations, B represents the convergence angle, C is the convergence angle, and D is the throat diameter. According to the coefficients of the coded equations, it can be said that in all these equations, the variable that has the greatest impact on each of the dependent variables of this research is the diameter of the throat.

Model adequacy was verified through ANOVA and residual analysis. Adjusted R² and lack-of-fit results confirmed good model performance, and no problematic multicollinearity was observed among the independent variables.

### 3.2. The effect of throat length, divergence angle, convergence angle, and throat diameter on TKE (max and ave)

The impact of the length and diameter of the venturi tube on TKE in a venturi tube reactor is highly significant, as the dimensions of this part of the reactor affect flow characteristics, mixing efficiency, and energy dissipation.

**Fig 3:**
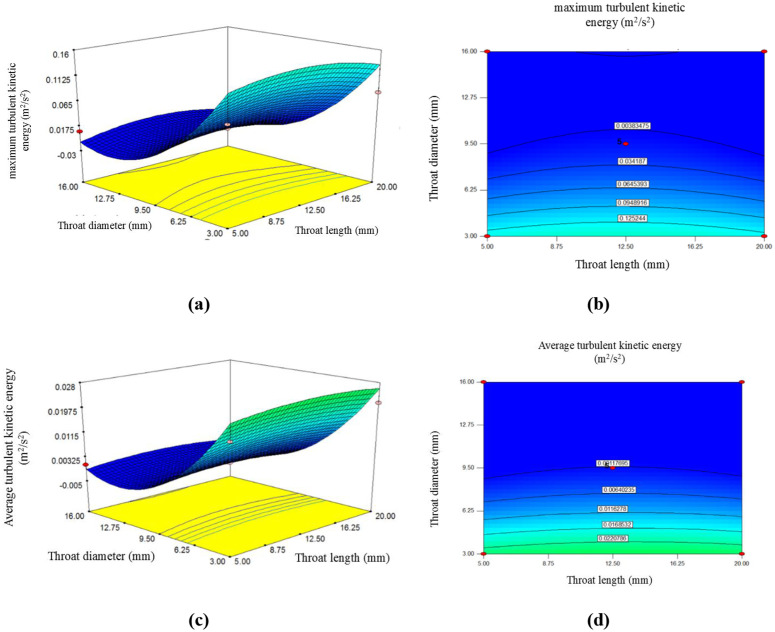
The effect of throat length and throat diameter on maximum turbulent kinetic energy: a) 3-D surface plot; b) contour plot; and average turbulent kinetic energy: c) 3-D surface plot; d) contour plot.

The Fig 3a shows the trend of maximum TKE changes as a function of throat length and diameter. As shown in Fig 3a, the maximum TKE inside the reactor increases gradually with the increase in throat length. Additionally, based on this figure, it is clear that increasing the diameter reduces TKE max. According to this figure, if the throat length increases from 5 mm to 12.5 mm, the TKE max changes from 0.0045 to 0.013 (m²/s^2^), indicating that increasing the throat length effectively raises the TKE max. However, if the throat length increases to 20 mm, the TKE max decreases slightly, approaching 0.006 m²/s^2^. This indicates that increasing the throat length has a positive effect on TKE max only up to a certain point, after which further increases lead to a decline, which is undesirable.

As shown in this figure, increasing the throat diameter of the venturi tube has the opposite effect on the increase in TKE max. The steep slope in this figure and the coefficient of the coded Equation (19) demonstrate that, among other independent variables, the throat diameter significantly impacts TKE max. When the throat diameter is considered to be 3 mm, the TKE max will be 0.15 m²/s². For a larger diameter, such as 9.5 mm, the maximum energy decreases to 0.013 m²/s². Further increasing the diameter to 16 mm leads to a continued decline in TKE max, reaching 0.006, which is undesirable.

**Fig 4:**
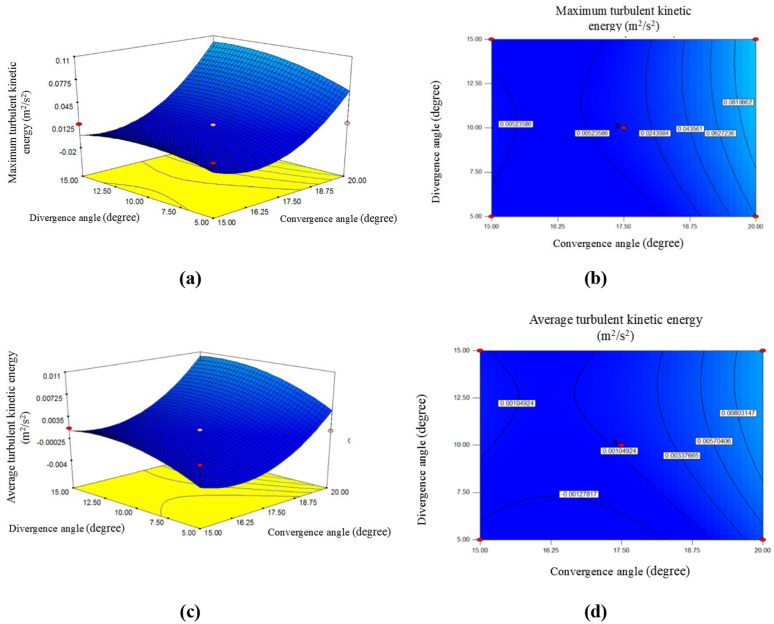
The effect of divergence angle and convergence angle on maximum turbulent kinetic energy: a) 3-D surface plot; b) contour plot; and average turbulent kinetic energy: c) 3-D surface plot; d) contour plot.

Fig 3c shows the TKE ave along the venturi tube. The TKE ave indicates turbulence and instability throughout the venturi tube, and the higher it is, the greater the mixing and dispersion on the surface of the tube. As Figs 3c and 4c indicate, this parameter is influenced by changes in variables such as throat length and diameter, as well as the convergence and divergence angles of the venturi tube.

Based on Fig 3c, increasing the throat length and diameter leads to a decrease in the TKE ave along the tube. In other words, the larger the throat length and diameter of the venturi tube, the lower the TKE ave. According to this figure, it is clear that changing the size of the throat diameter is much more effective in influencing the TKE ave. Also, the coefficient of coded Equation (20) also shows that, changes in the diameter of the throat have an inverse effect on TKE ave. And as the throat diameter increases, the average of this energy decreases. According to the figure, when the throat length is considered to be 5 mm, the TKE ave is 0.0006 m²/s^2^. If the reactor has a larger throat length, for example, 12.5 mm, the TKE ave increases to 0.0013 m²/s^2^. As the figure shows, increasing the throat length beyond this point is undesirable. It leads to a decrease in the TKE ave for example, a throat length of 20 mm results in an TKE ave of 0.0002 m²/s^2^.

As shown on this figure, the decrease in TKE ave with increasing throat diameter occurs at a steep slope. A detailed figure analysis reveals that when the throat diameter is 3 mm, the TKE ave is 0.027. If the venturi tube has a larger diameter, such as 9.5 mm, this energy decreases significantly to 0.0013 m²/s^2^. To better examine this, a tube with an even larger diameter of 16 mm was tested, showing that the TKE ave continued to decrease, reaching 0.0005 m²/s^2^. Thus, it can be concluded that a smaller throat diameter in a venturi tube leads to higher and more favorable TKE ave.

Studies show that increasing the converging section length of a venturi tube enhances TKE in the throat area of the venturi tube reactor. This is because a longer convergence allows for more focused energy, increasing turbulence and the potential for energy transfer. However, this effect diminishes at the throat exit and in the diverging section, where pressure recovery and reduced flow velocity decrease TKE [24]. Also it should be noted that if the throat length is too short, the flow does not fully develop, which limits the generation of turbulent flow [25]. Research conducted by (Zheng et al., 2021) found that a longer throat in a venturi tube reactor results in more stable flow, reduced turbulence, and a more uniform velocity profile.

Previous studies also show that the throat length of a venturi tube significantly affects the removal of microorganisms by TKE and inducing cavitation. These studies indicate that throat length impacts the cavitation number and flow velocity inside the tube, which, in turn, influences the rate of pathogen inactivation. Research [26] revealed that the throat length of a venturi tube should be optimized, as an excessively long throat does not yield desirable results. Their findings suggested that shorter throats, compared to longer ones, increase the effects of TKE and cavitation, which enhances microbial cell wall damage through microjets and shock waves.

The diameter of the venturi tube, particularly in the throat section, is critical in determining flow velocity and turbulence levels. A smaller throat diameter increases flow velocity, resulting in increased TKE. However, excessive reduction in diameter can lead to excessive cavitation and energy losses, which may negate the beneficial effects of increased turbulence and flow mixing. Conversely, increasing the throat diameter reduces flow velocity, thereby decreasing TKE, which makes it less effective for processes that require increased turbulent flow [25,27].

Results from many studies have shown that the throat diameter of a venturi tube significantly affects TKE during the pasteurization process. A smaller throat diameter typically leads to increased fluid velocity and greater pressure variations, which can enhance TKE and cavitation. This improves mixing efficiency and mass and heat transfer, which are essential for the pasteurization process [28]. In a study conducted by [29], a venturi tube reactor with different throat diameters was constructed for waste water treatment. The results showed that a venturi tube with a 3 mm throat diameter could generate more microbubbles than 4 mm and 5 mm diameters.

In conclusion, optimizing the throat length and diameter of a venturi tube reactor requires balancing increased turbulent flow with minimal energy losses and achieving maximum TKE. A well-designed venturi tube reactor ensures that turbulence is efficiently and effectively generated, which is crucial for applications requiring mixing and turbulence creation.

The convergence and divergence angles of a venturi tube reactor significantly affect TKE, as these angles influence flow characteristics, pressure distribution, and cavitation behavior. Fig 4a shows the impact of TKE max under the influence of the convergence and divergence angles of the venturi tube. Based on the trends of the slopes, it is evident that as the convergence and divergence angles increase, the TKE max within the tube also increases. Additionally, considering the steep slope that shows in this figure and the codded equation coefficient [19], it can be concluded that changes in the convergence angle have a greater effect on variations in TKE max. Moreover, as the figure shows, increasing the convergence angle increases the TKE max throughout the tube. For example, when the convergence angle is 5˚, the TKE max is approximately 0.010 m²/s². As this angle increases to 17.50˚, the TKE max becomes even higher, reaching 0.014 m²/s². In fact, it can be concluded that as the convergence angle increases, the TKE max also rises. The figure shows that at a convergence angle of 20˚, the TKE max reaches 0.093 m²/s².

Conversely, increasing the divergence angle has a smaller effect on TKE max changes. When the divergence angle is 5˚, the TKE max is 0.007 m²/s^2^. If the divergence angle is increased slightly to 10˚, the TKE max increases slightly, approaching 0.013 m²/s^2^. If examine the TKE max at a larger angle, such as 20˚, the TKE max decreases slightly to 0.011 m²/s².

The Fig 4c also illustrates the changes in TKE ave under varying convergence and divergence angles. As the figure shows, increasing these angles increases the TKE ave, with the convergence angle having a more pronounced effect on increasing the TKE ave. If the Venturi tube’s convergence angle is considered 15˚ or 17.50˚, the TKE ave values are 0.001352 and 0.001354 (m²/s^2^), respectively, indicating that these two values show no significant difference. But, if the convergence angle increases to around 20˚, the TKE ave reaches 0.0093 m²/s^2^, demonstrating that a larger convergence angle is needed to achieve a higher TKE ave.

On the other hand, it can be said that a smaller divergence angle, such as around 5˚, results in TKE ave of 0.0028 m²/s^2^. As the angle increases to 10˚, the TKE ave increases to 0.0013 m²/s^2^. Further examination at a divergence angle of 15˚ shows a continued upward trend, with the TKE ave approaching 0.0017 m²/s^2^. Thus, it can be concluded that as the divergence angle increases, the TKE ave also increases. It is evident that limitations accompany this increasing trend.

Previous studies have reported that a sharper convergence angle (smaller) increases the speed in the throat more rapidly, resulting in a stronger flow acceleration and an increase in TKE. This increase in velocity leads to more turbulence in the throat region, where mixing and energy dissipation are most important. As these studies also show, if the convergence angle is too small or sharp, it can cause flow separation, leading to inefficiency and a reduction in effective TKE downstream. In fact, the optimal convergence angle should create a balance between acceleration velocity and preventing flow separation [30].

The divergence angle of the venturi tube primarily affects pressure recovery and flow stabilization after the throat. A smaller or sharper divergence angle allows for smoother pressure recovery and reduces energy loss, which can help maintain TKE in the downstream sections of the flow. If the divergence angle is too small, it may result in a longer pressure recovery process, reducing the maximum TKE generated in the throat [27,30]. Conversely, it should be noted that if the divergence angle increases, it enhances the breakdown and destruction of turbulent vortices, leading to greater energy dissipation and increased mixing. However, if this angle becomes excessively large, it can create undesirable pressure gradients, flow separation, and a reduction in TKE [25,27,30].

In general, from the analysis of this figure, it can be concluded that the convergence angle within the venturi tube reactor controls the acceleration, velocity, and TKE max in the throat region of the tube. In contrast, the divergence angle affects energy dissipation, pressure recovery, and TKE dissipation. Therefore, the size of both angles must be optimized to ensure maximum turbulence occurs within the tube under optimal conditions without causing excessive flow separation or energy dissipation.

A study (Pegu, More, & Arya, 2023) used a hydrodynamic cavitation reactor to pasteurize milk to eliminate *E. coli*. This study concluded that both the geometry of the hydrodynamic cavitation reactor and flow characteristics (flow velocity and cavitation number) play roles in TKE and subsequently in the occurrence of cavitation, thereby affecting the reduction of bacteria. Therefore, hydrodynamic cavitation, with proper design, can be a potential option for non-thermal food pasteurization.

### 3.3. The effect of throat length, divergence angle, convergence angle, and throat diameter on TED rate

**Fig 5:**
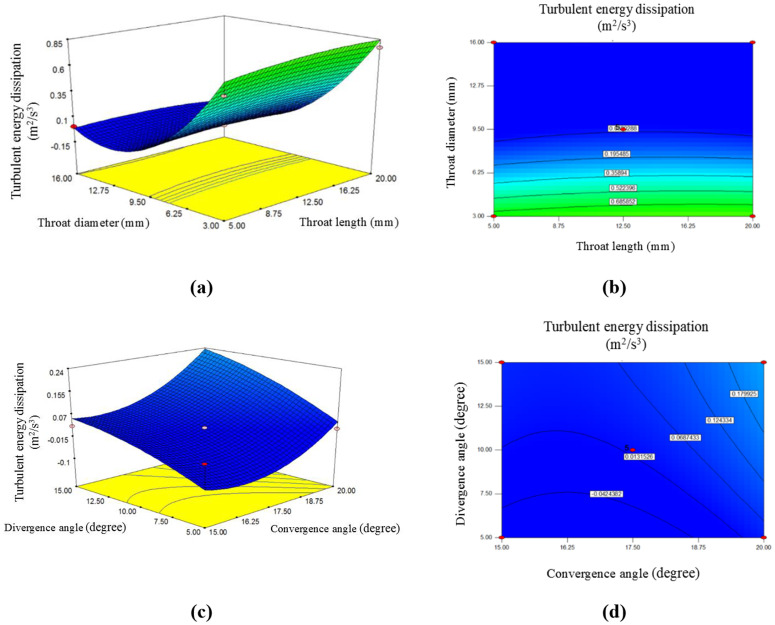
The effect of throat length, divergence angle, convergence angle, and throat diameter on turbulent energy dissipation: a, c) 3-D surface plot; b, d) contour plot.

**Fig 6:**
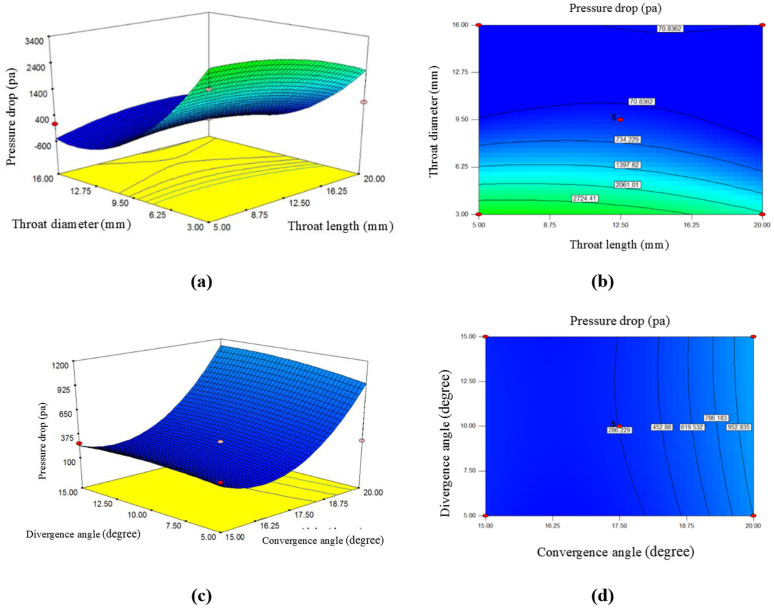
The effect of throat length, divergence angle, convergence angle, and throat diameter on pressure drop: a, c) 3-D surface plot; b, d) contour plot.

The throat length, venturi diameter, convergence angle, and divergence angle are four key parameters influencing the TED rate. The TED rate is one of the most critical parameters in turbulent flow modeling, as it represents the amount of kinetic energy from turbulence converted into heat due to viscous forces. Since energy dissipation is generally undesirable, the goal in this section is to minimize this variable. Figs 5 and 6 illustrate the average TED resulting from changes in throat length and diameter and the venturi tube’s convergence and divergence angles.

Fig 5a shows the throat length and diameter effect on TED rate. It is clear that the average TED decreases with an increase in throat length and diameter. However, based on the slope that shows in this figure, it can be said that changes in throat diameter have a significantly greater effect on changes in average TED. According to this figure, in a venturi tube with a throat length of 5 mm, the TED rate is approximately 0.028 m^2^/s^3^. If the throat length slightly increases to 10 mm, the energy dissipation rate increases to about 0.019 m^2^/s^3^. For a better understanding of this issue, the throat length was increased to 20 mm, and it was observed that the energy dissipation rate further decreased to 0.004 m^2^/s^3^. A longer venturi throat increases the area where turbulence can develop, resulting in a greater overall energy dissipation due to turbulence.

As the fluid moves through the throat, energy transfer between vortices and viscous effects increases, leading to greater TED. Based on previous research results, it can be concluded that if the throat length becomes excessively long, this energy dissipation may become excessive, leading to a decrease in turbulence intensity downstream of the flow [24]. On the other side of Fig 5a, the changes in TED rate due to changes in throat diameter are shown. According to this figure, if the diameter is 3 mm, the TED rate is around 0.83 m^2^/s^3^. If the throat diameter slightly increases to about 9.5 mm, the TED rate decreases to approximately 0.019 m^2^/s^3^. To further examine this topic, a diameter of 16 mm was also considered, and it was determined that the TED rate decreased to 0.0022 m^2^/s^3^. Therefore, it can be concluded that as the throat diameter increases, the TED rate decreases. It is clear that limitations also accompany this phenomenon.

Previous research has shown that a smaller throat diameter significantly increases fluid velocity, enhancing the shear forces caused by turbulence, which, in turn, increases energy dissipation due to turbulence. This process transfers more energy from larger vortices to smaller scales, leading to greater energy dissipation in the form of heat [27,31].

On the other hand, larger diameters in the venturi tube reduce flow velocity, which leads to a decrease in TED. Therefore, an optimal diameter must be considered to balance high flow velocity for effective mixing and minimizing energy dissipation [18,19].

The Fig 5b also illustrates the trend of TED rate changes influenced by variations in convergence and divergence angles. According to this figure, the TED rate also increases with an increase in either the divergence or convergence angles. However, considering the trends of slopes and the coefficients from the coded Equation (21), it can be stated that changes in the divergence angle have a more significant impact on the TED rate compared to changes in the convergence angle.

The changes in the convergence angle indicate that the convergence angle (especially small convergence angles) causes a rapid acceleration of the fluid, increasing turbulence near the throat. This sudden acceleration enhances the energy transfer process and consequently increases TED, particularly in the throat region [32].

If the convergence angle is excessively steep, it may cause flow separation, resulting in energy dissipation due to turbulence breakdown, which can negatively impact the efficiency and performance of this system [33].

According to this figure, when the convergence angle is 15˚, the TED rate is 0.011 m^2^/s^3^. If this angle increases (to 17.5˚), the amount of energy dissipation increases to 0.019 m^2^/s^3^. If this angle increases further to 20˚, the TED rate rises, approaching 0.16 m^2^/s^3^.

This figure shows the trend of changes in the divergence angle and its effect on the TED rate, it is clear that as this angle increases, the TED rate rises. Therefore, when the divergence angle is 5˚, the energy dissipation rate is 0.019 m^2^/s^3^, and in a scenario where this angle increases by another 5˚, the energy dissipation rate also rises to 0.078 m^2^/s^3^. If this angle is set to 15˚, the dissipation rate will again increase to 0.084 m^2^/s^3^.

In fact, small divergence angles facilitate gradual pressure recovery and reduce the likelihood of flow separation, providing better control over TED. This process contributes to a more uniform energy distribution without excessive TED [34].

In contrast, a larger divergence angle increases TED because flow separation and vortex formation result in higher energy loss. This condition leads to greater TED, which can decrease the overall reactor efficiency [35].

In summary, TED is significantly influenced by the geometry and dynamics of flow in the venturi tube reactor. By optimizing these parameters, it is possible to manage energy dissipation and maximize mixing efficiency within the tube.

### 3.4. The effect of throat length, divergence angle, convergence angle, and throat diameter on pressure drop

The parameters such as throat length and diameter, as well as the convergence and divergence angles of the venturi tube, affect the flow dynamics and energy dissipation within the system, and they also have a significant effect on the pressure drop throughout the reactor.

The diagrams in Figs 6a and 6c demonstrate the influence of the independent variables, namely throat length, throat diameter, divergence angle, and convergence angle, on the dependent variable, which is the pressure drop. The diagram in Fig 6a illustrates the variations in pressure drop as influenced by throat length and diameter changes. According to this diagram, increasing either of these parameters results in a reduction in the pressure drop. Based on the slope of the two sections of the diagram and the coefficients of the codded Equation (22), it can be observed that the throat diameter has a more significant effect on pressure drop compared to the changes in throat length. To better analyze this, according to the diagram (6a), when the throat diameter is 3 mm, the pressure drop is approximately 3115.13 Pa. When a venturi tube reactor with a throat diameter of 9.50 mm is used, this pressure drop decreases to 290.241 Pa. If the throat diameter is further increased to 16 mm, the pressure drop reduces even more, reaching 113.65 Pa.

This trend shows that as the throat diameter increases, the pressure drop decreases. The negative coefficient of the throat diameter variable in the codded equation (Equation 22) further confirms this, indicating that a larger throat diameter leads to lower pressure drop within the venturi tube. In other words, the larger diameter of the venturi tube reduces the velocity and leads to a lower pressure drop, but at the cost of reducing the TKE and less effective mixing of the fluid, which is not a desirable situation [36].

To examine the effect of the throat length of the venturi tube, according to the diagram (6a), it is evident that the increasing trend of pressure drop with throat length is limited. If the throat length of the tube is 5 mm, the pressure drop will be around 188.321 Pa. However, if the tube is designed with a longer length, 12.50 mm, this pressure drop increases to 290.241 Pa compared to the previous length. However, as previously mentioned, this trend does not always continue to increase and is subject to limitations. For example, when the throat length is 20 mm, the pressure drop decreases to 231.31 Pa.

A longer venturi throat can lead to a greater pressure drop due to prolonged fluid interaction with the walls, causing more friction and energy dissipation. Essentially, the longer the venturi throat, the more viscous forces and shear stresses are generated and developed, increasing pressure drop [32]. It should also be considered that reducing the throat length can decrease friction losses, but it may also affect the ability to generate turbulence and turbulent flow [37].

Two other important variables to consider are the convergence and divergence angles. As shown in diagram (6c), changes in the convergence angle occur with a steeper slope than changes in the divergence angle, indicating that the convergence angle has a more significant impact on the pressure drop within the venturi tube.

According to the diagram, when the reactor is designed with a convergence angle of 15˚, the pressure drop is 280.93 Pa. If this angle is increased slightly to about 17.50˚, the pressure drop increases by approximately 3%, reaching 290.241 Pa. When the convergence angle is further increased to 20˚, the pressure drop within the venturi tube continues its upward trend, reaching 1080.21 Pa. Analyzing this diagram reveals that increasing the convergence angle of the venturi tube leads to an increase in the pressure drop. However, previous studies have shown that small convergence angles cause rapid fluid acceleration, resulting in a more significant pressure drop in the throat section of the venturi tube. Nevertheless, if the angle is too small or too steep, the likelihood of flow separation increases, leading to greater energy dissipation and higher pressure drop [38].

On the left side of the diagram (6c), which shows the pressure drop changes due to the divergence angle, it is shown that when the divergence angle is 5˚, the average pressure drop is 203.21 Pa. When a reactor with a larger divergence angle (10˚) is used, the average pressure drop increases slightly compared to the previous angle, reaching 290.241 Pa. If the divergence angle is further increased to 15˚, the increase in average pressure drop occurs much slower, reaching 291.296 Pa. Therefore, based on the analysis of this diagram, it can be concluded that increasing the divergence angle has a negligible effect on the average pressure drop, resulting in a very mild increase in the average pressure drop.

A small divergence angle allows for smoother pressure recovery and consequently reduces the overall pressure drop after the throat, leading to a more uniform flow distribution and minimizing energy dissipation. Conversely, a larger divergence angle can increase the pressure drop due to more significant energy losses and potential flow separation in the diffuser section. The larger the angle, the more difficult it is for the flow to recover, resulting in a higher pressure drop [38]. Overall, the analysis of these diagrams showed that the pressure drop is highly sensitive to the mentioned parameters, and balancing these parameters is crucial for achieving efficient pressure recovery, minimizing energy dissipations, and optimizing the performance of the venturi tube reactor.

In a study by [39], they used a hydrodynamic cavitation reactor to treat and eliminate both Gram-positive and Gram-negative bacteria from various water sources. Their findings revealed that the reactor’s geometry significantly influenced the disinfection and sanitation processes, and it was determined that a significant pressure drop condition (10 bar) is necessary for effective disinfection and high-efficiency bacterial removal.

### 3.5. The effect of throat length, divergence angle, convergence angle, and throat diameter on average VD

**Fig 7:**
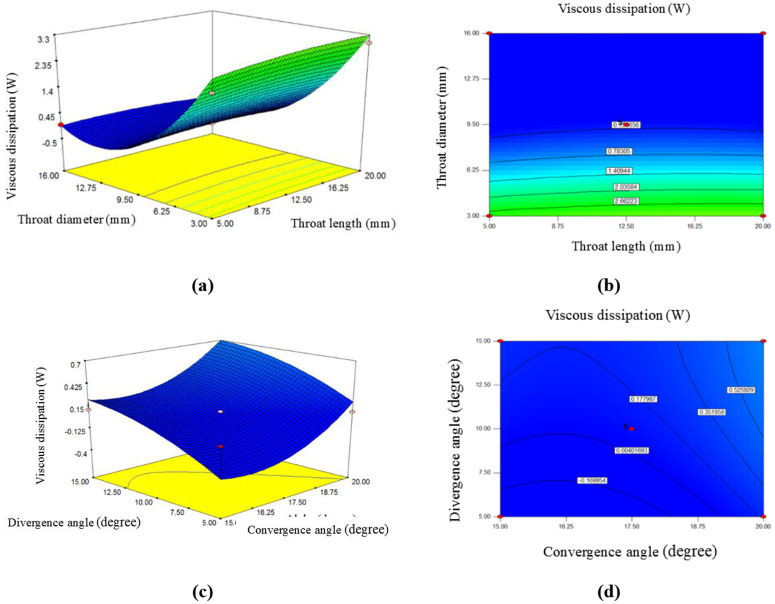
The effect of throat length, divergence angle, convergence angle, and throat diameter on viscous dissipation: a, c) 3-D surface plot; b, d) contour plot.

Fig 7 shows the effects of changing some reactor dimensions, including the throat length and diameter, as well as the convergence and divergence angles of the venturi tube, on the average VD.

As the slope in this figure indicates, the effect of the throat diameter and divergence angle on the increase or decrease of the average VD is more significant than the other independent variables. Additionally, based on the coefficients in the codded Equation (23), it can be concluded that the throat diameter has the most substantial effect on the average VD among the four independent variables. According to Equation (23) the negative sign of the coefficient for this variable indicates that it has an inverse effect on the average VD, meaning that as the throat diameter increases, the average VD decreases.

According to the figure showing the effect of throat diameter and length, it can be observed that increasing the throat length in the venturi tube results in an increase in the average VD inside the tube. This trend is limited; if the throat length exceeds a certain limit, the average VD decreases. Three lengths of 5 mm, 12.50 mm, and 20 mm were considered for the throat length.

The results showed that the average VD for each length was 0.081 W, 0.086 W, and 0.019 W, respectively, demonstrating the increasing trend with limitations. A longer throat section typically increases the interaction between fluid layers, leading to greater average VD. Specifically, in this case, a longer throat section allows more time for the high-velocity core of the flow to transfer energy to surrounding layers via viscous forces, resulting in greater energy dissipation [40].

Therefore, it can be interpreted that having a long throat section in this reactor could reduce the overall efficiency, as the energy dissipation may lower the TKE before reaching the target areas, thus diminishing the reactor’s desired effects. On the other hand, the throat diameter is the most important and influential parameter in changing the average VD. In this figure, the changes in throat diameter are shown at three levels: 3 mm, 9.50 mm, and 16 mm. As the figure indicates, increasing the throat diameter of the venturi tube decreases the average VD.

The results show that if these three diameters are used in constructing a venturi tube reactor, the average VD for each diameter from smallest to largest would be 3.21 W, 0.086 W, and 0.025 W, respectively.

As previous research has shown, a smaller throat diameter in the venturi tube increases flow velocity, increasing turbulence and VD. In such a case, shear forces, especially near the walls, become more intense, contributing to greater energy dissipation through viscosity [18]. Conversely, a larger throat diameter reduces the velocity, resulting in lower VD, but it should be noted that it also reduces the TKE, which may affect mixing efficiency within the venturi tube [19].

Fig 7c shows the effect of changes in the divergence and convergence angles of the venturi throat on the average VD. As the slope in the divergence angle section suggests, this parameter has a greater influence on average VD than the convergence angle. From this figure, it is clear that increasing the convergence angle also increases the average VD. When this venturi tube is constructed with convergence angles of 15°, 17.50°, and 20°, the corresponding average VD values are 0.062 W, 0.086 W, and 0.55 W, respectively. Similarly, when the divergence angle is tested at three levels: 5°, 10°, and 15°, the average VD values are 0.27 W, 0.086 W, and 0.24 W, respectively.

The trend of this figure indicates that increasing the convergence and divergence angles of the venturi tube leads to an increase in average VD. A steep or small convergence angle accelerates the flow rapidly, increasing velocity gradients and enhancing VD near the throat due to more intense shear forces. However, if the angle is too steep, it may cause flow separation, leading to energy dissipation and reduced reactor efficiency. In contrast, creating a more moderate angle provides smoother acceleration, producing more controlled VD and optimizing performance by balancing energy dissipation with energy conservation.

On the other hand, a smaller divergence angle helps gradually recover pressure and minimizes flow separation, leading to controlled VD. This helps preserve the TKE in the downstream flow. In contrast, a larger divergence angle increases turbulence and leads to a greater breakdown in velocity gradients, resulting in increased VD and overall energy loss. Flow separation may occur if the angle is too large, leading to significant energy loss. Optimizing these four parameters is crucial for managing VD and ensuring the proper performance of the venturi tube reactor, especially in processes like this study, where minimizing energy loss is essential for maximizing fluid mixing and reaction rates. Research conducted by [41] indicates that VD increases fluid temperature, which helps improve the pasteurization process and eliminates microorganisms.

### 3.6. Process optimization

In this study, optimization was carried out using the RSM in the Design Expert software to enhance TKE, increase the TKE ave, TKE max, reduce the rate of TED, minimize pressure drop, and reduce average VD.

The four independent variables in this experiment, including the throat length (L_th_), convergence angle (β), divergence angle (α), and throat diameter (d_th_), can be adjusted within specified ranges and based on the boundary conditions. Essentially, this optimization aims to determine the input parameter conditions that lead to an increase in TKE and a reduction in the dissipation rates within the venturi tube reactor. The optimization revealed that when the throat diameter and length of the tube are set at 4.91 mm and 13.15 mm, respectively, and the convergence and divergence angles are set at 19.2˚ and 8.31˚, respectively, the TKE and its average are 0.2438 m^2^/s^2^ and 0.0305 m^2^/s^2^, respectively. Moreover, the TED rate, pressure drop, and reduction in average VD were found to be 0.7151 m^2^/s^3^, 3483.01 Pa, and 2.69 W, respectively.

**Fig 8:**
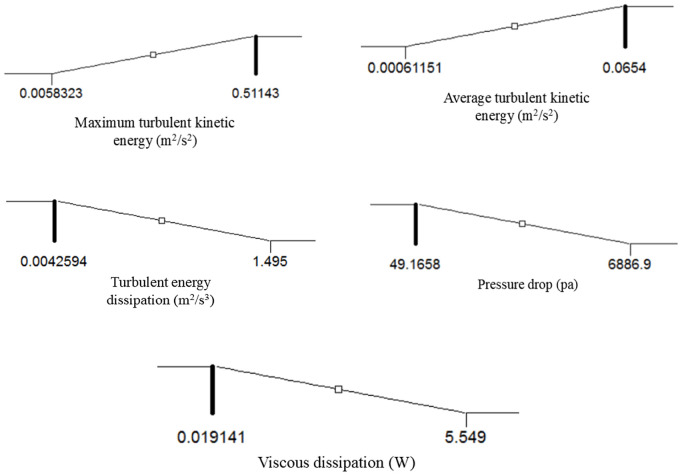
Optimum conditions predicted by RSM method.

The proposed point and optimal conditions were subsequently simulated, demonstrating that under these conditions, the TKE is 0.3046 m^2^/s^2^, the TKE ave is 0.034 m^2^/s^2^, the TED rate is 0.6951 m^2^/s^2^, the pressure drop is 3835.05 Pa. The average VD is 2.86 W (Fig 8).

**Fig 9:**
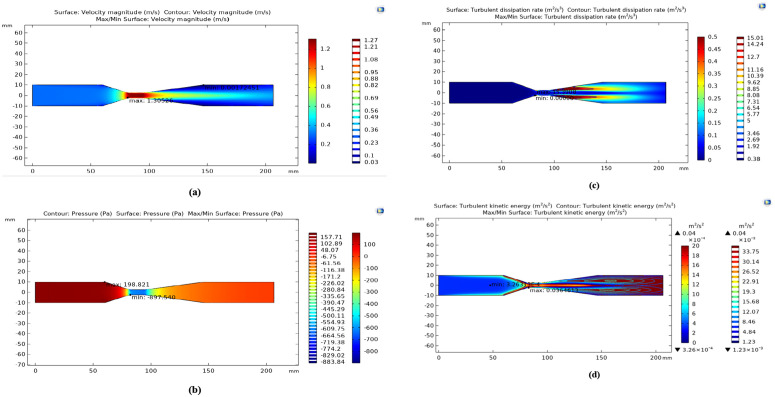
Contour of: a) velocity, b) pressure, c) turbulent kinetic energy, and d) turbulent energy dissipation under optimal conditions.

After obtaining the optimal point of this experiment using Design Expert software and the RSM, the venturi tube reactor was simulated under these optimal conditions using CFD methods. Fig 9 shows the contour of velocity, pressure, TKE, and TED variations inside the tube under optimal conditions.

Fig 9a illustrates the velocity distribution of the fluid along the venturi tube. In this Figure, the tube’s inlet is on the left, and the outlet is on the right. The velocity at the tube’s entrance and inlet section ranges from 0.2 m/s to 0.4 m/s. Based on this Figure, it can be said that the velocity is uniform at the beginning of the tube and before reaching the converging section of the venturi, indicating laminar flow inside the tube. Additionally, from the color scale of this figure, it is clear that the velocity near the tube walls (dark blue regions) is very low and almost close to zero. This low velocity is due to the no-slip condition assumed at the tube walls during the simulation. As soon as the fluid flow enters the venturi throat, the velocity increases, and the highest velocity occurs along the throat, reaching 1.3053 m/s. This velocity increase in this section of the tube can be explained by Bernoulli’s principle, where a decrease in the tube diameter and a pressure drop lead to an increase in velocity.

After the fluid passes through the throat and moves towards the diverging section of the venturi tube, where the diameter increases again, the fluid velocity gradually decreases. The velocity in this region ranges from 0.9 m/s to 0.4 m/s. In fact, it can be concluded that after passing through the throat and with the subsequent increase in diameter, the fluid nearly regains its initial velocity.

Fig 9b shows the pressure distribution from the inlet to the venturi tube outlet. According to this diagram, pressure varies at different points along the tube, following Bernoulli’s principle. This diagram shows that the maximum pressure occurs at the inlet and early section of the tube, with a value of 198,821 Pa. This maximum pressure results from the larger tube diameter in this region, as under such conditions, the fluid velocity decreases, leading to a pressure increase. There is a significant pressure drop after the fluid passes this section and reaches the converging throat.

As previously mentioned, the reduction in diameter at this section causes an increase in velocity and a decrease in pressure. It can even be observed that in this section, the pressure reaches a negative value of −897.540 Pa. The pressure drop in the throat becomes so significant that the local fluid pressure may fall below its vapor pressure, increasing the risk of cavitation. As seen in the diverging section near the outlet, the pressure gradually recovers as the diameter approaches the inlet size. Still, it does not return to its initial pressure due to the dissipations that occur within the venturi tube during this process.

Fig 9c shows the TKE variations along the venturi tube. The results show that the maximum TKE is approximately 0.03648 m²/s², observed in the venturi throat. This region exhibits significant turbulence due to sudden changes in cross-sectional area and increased fluid velocity. In contrast, areas before entering and after exiting the narrow section exhibit lower TKE. The figure shows that the minimum TKE value is 3.2631 × 10 ⁻ ⁴ m²/s², indicating a calmer flow and lower fluid velocity in this section. The TKE distribution is symmetric along the tube, with the highest concentration in the flow center and the narrow section of the venturi. Analyzing this parameter is crucial for evaluating mixing quality, heat and mass transfer efficiency, and pressure drop in various systems, including pasteurization reactors.

Fig 9d provides a detailed analysis of the TKE rate. This parameter represents the energy dissipated as heat or other non-useful forms due to flow turbulence. The fluid velocity increases significantly in the venturi throat due to the reduced cross-sectional area. This increase in velocity creates strong flow turbulences, resulting in a rise in the TED rate. According to the data, this region’s maximum TED reaches approximately 15.391 m²/s³. This high dissipation indicates intense vortices form here, and a significant portion of the fluid’s energy is lost due to turbulence and eddies. The fluid flow gradually calms down after passing through the narrow section and entering the diffuser region.

The decrease in fluid velocity and the reduction in sudden changes in cross-sectional area cause turbulence to subside, resulting in a lower TED rate. In this section, the minimum dissipation rate becomes so small that it approaches zero, indicating a very calm flow with minimal energy loss.

These changes in the turbulent energy dissipation rate illustrate the flow behavior within a venturi system, where turbulence and energy dissipation increase significantly in the throat and then decrease in the diffuser region. From the analysis of these figures, it can be concluded that the velocity increase and pressure reduction in the throat section contribute to improved mixing and heat transfer. This process can enhance the efficiency of pasteurization and lead to a more effective reduction of microorganisms.

Furthermore, the turbulence generated by the flow in the throat aids in heat generation and temperature rise, which are key factors in microorganism elimination. Thus, the design and application of venturi tubes in pasteurization systems can positively impact product quality and safety. Additionally, the results obtained from the software completely agree with Bernoulli’s principle and previous studies, affirming that the simulation and the reactor perform effectively and correctly.

### 3.7. Correlation analysis between dependent variables

In this section, Pearson correlation analysis between the dependent variables of the current study was conducted using Minitab software. The presented correlation diagrams show the statistical relationship between the variables using Pearson’s correlation coefficient (R).

**Fig 10:**
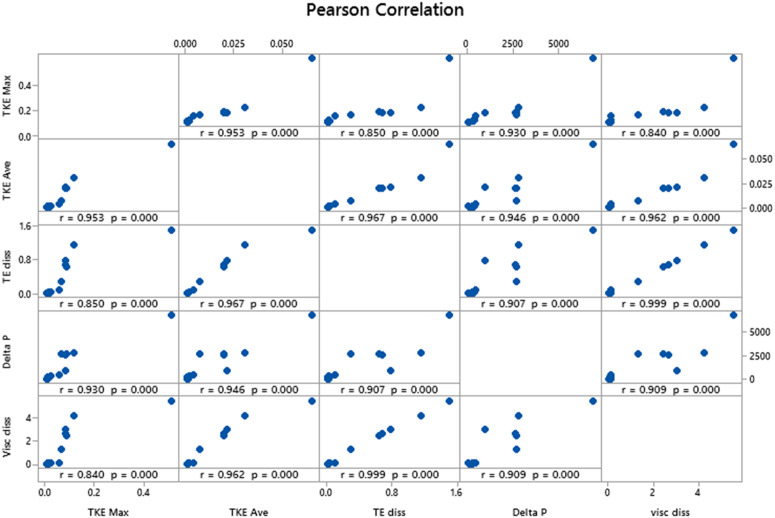
Correlation analysis of dependent variables.

The correlation coefficient is a number between −1 and +1, where a value of +1 indicates a perfect positive correlation, 0 indicates no correlation, and −1 indicates a perfect negative correlation between the examined variables. The value “p” represents the p-value between each pair of variables, and if this value is less than 0.05, it indicates a statistically significant relationship between the two variables. According to Fig 10, and based on the results of Pearson’s correlation, strong and significant statistical relationships are observed between the dependent variables. As shown in Fig 10, variables such as TKE max, TKE ave, TED rate, pressure drop, and average VD all have strong positive correlations.

This suggests that a strong interdependent relationship exists between the experimental variables, indicating that they have synergistic effects on one another. Very strong correlations (especially those greater than 0.9) indicate that changes in one variable are highly likely to cause similar changes in another variable, which could be an important factor to consider in future simulations.

One of the highest correlation coefficients in this analysis is the correlation between pressure drop and mean VD, which is 0.999. This value indicates an extremely strong and nearly perfect positive relationship between these two variables. Additionally, the p-value is 0.00001, confirming that this relationship is statistically significant. In other words, it can be said that changes in pressure drop within the venturi tube almost directly affect the mean VD.

Moreover, as seen in Fig 10, the correlation between TKE max and mean VD is 0.840. This value reflects a strong positive correlation between these two variables, although the intensity of the correlation is slightly lower compared to other variables. The p-value of 0.00001 further confirms this correlation’s statistical significance and importance between the two dependent variables.

#### 3.7.1. Experimental validation of the CFD results.

Based on the CFD simulations and the optimized reactor dimensions obtained in the previous sections, a validation step is required to ensure that the hydrodynamic predictions are consistent with real operational performance. For this purpose, a reactor with the optimized geometry was fabricated and used for non-thermal pasteurization experiments. The microbial reduction results presented in the following sections (3.8–3.9) provide experimental evidence that supports and validates the simulation outcomes, confirming the reliability of the proposed reactor design and its predicted flow characteristics.

### 3.8. Experimental results of milk pasteurization using a venturi tube reactor

Experimental pasteurization of cow milk was performed in the laboratory using venturi tube reactor to validate the simulations. According to the results obtained in the previous sections, it was found that increasing the TKE leads to increased mixing, cavitation, and agitation inside the reactor, and as a result, pasteurization and destruction of microorganisms are performed much better by increasing the TKE inside the venturi tube. increasing the TKE and cavitation, enhances microbial cell wall damage, also by increasing the TKE, cavitation number and flow velocity inside the tube, can influences the rate of pathogen inactivation. As mentioned in the previous sections this improves mixing efficiency, mass and heat transfer, which are essential for the pasteurization process. Therefore, it can be concluded that this method can be a potential option for non-thermal food pasteurization.

The reduction of *Escherichia coli* (*E. coli*) in milk is essential for ensuring food safety. The results confirmed that higher TKE levels improved the disruption of microbial cell walls, particularly for *E. coli*, consistent with the simulation predictions. In a study conducted by [42], investigated that the elimination of *E. coli* bacteria through the implementation of a hydrodynamic cavitation system composed of static mixers, Venturi tubes and perforated plates. This study reveals an important biocidal power, which makes it very effective in the purification and disinfection of liquids. Also this stude showed that bacterial cell membranes faced weakening and death due to the combined mechanical and chemical effects of cavitation. These experimental results confirm the validity of the CFD predictions.

#### 3.8.1. Correlation between TKE and *E. coli* reduction.

**Fig 11:**
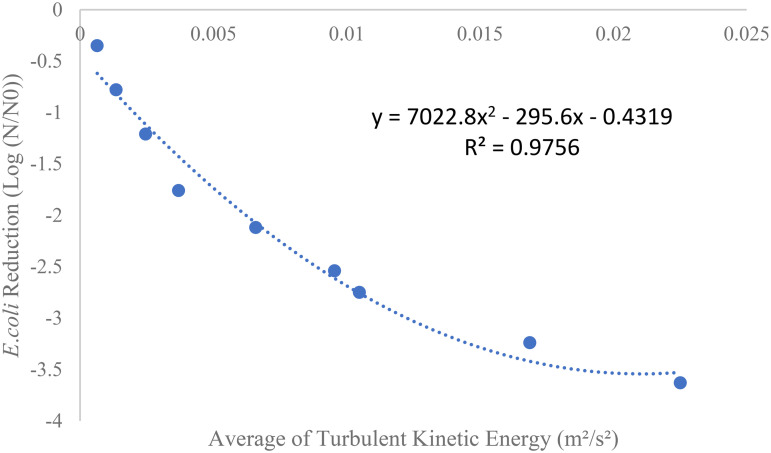
Relation between Turbulent kinetic energy and *E.coli* reduction.

The experimental results indicate a strong relationship between *Escherichia coli* (*E. coli*) reduction and the turbulent kinetic energy (TKE) generated in the Venturi tube reactor. The analysis revealed that as TKE increases, microbial inactivation efficiency improves significantly (Fig 11). This relationship is not linear but quadratic, implying that microbial inactivation is more pronounced at higher turbulence levels but only up to a certain point.

The quadratic equation that best describes this relationship is:


$$\begin{array}{cc} y=\text{7}\text{0}\text{2}\text{2}.\text{8}x^{\text{2}}-\text{2}\text{9}\text{5}.\text{6}x-\text{0}.\text{4}\text{3}\text{1}\text{9}(R^{\text{2}}=\text{0}.\text{9}\text{7}\text{5}\text{6})y=\text{7}\text{0}\text{2}\text{2}.\text{8}x^{\text{2}}-\text{2}\text{9}\text{5}.\text{6}x-\text{0}.\text{4}\text{3}\text{1}\text{9}\backslash quad(R^{\text{2}}=\text{0}.\text{9}\text{7}\text{5}\text{6})\\ & y=\text{7}\text{0}\text{2}\text{2}.\text{8}x^{\text{2}}-\text{2}\text{9}\text{5}.\text{6}x-\text{0}.\text{4}\text{3}\text{1}\text{9}(R^{\text{2}}=\text{0}.\text{9}\text{7}\text{5}\text{6}) \end{array}$$

Where y represents the log reduction of *E. coli* (Log(N/N₀)), x represents the TKE, R² = 0.9756 indicates a very strong fit between the observed data and the model, suggesting that the equation accurately describes the trend.

This quadratic correlation shows that as the TKE generated in the reactor increases, the *E. coli* reduction efficiency also rises. Specifically, the maximum reduction is achieved at an optimal point of TKE, beyond which the benefits of further increasing TKE begin to plateau. The turbulence and cavitation effects generated by high TKE values intensify the disruption of bacterial cell membranes, leading to higher rates of microbial inactivation. In particular, cavitation where tiny gas bubbles form and collapse creates intense localized forces that contribute to cell wall disruption, making it a key mechanism for inactivating *E. coli*. Moreover, the findings are supported by the CFD simulations, which helped determine the optimal TKE point for maximum *E. coli* inactivation. These simulations provided a valuable prediction model that guided the experimental setup and allowed for the identification of the most effective operating conditions for the reactor.

As mentioned in Fig 11 the quadratic trend shows that microbial inactivation improves as TKE increases, but only up to a critical point. As in the previous section, at the optimum point (throat diameter of 4.91 mm, tube length of 13.15 mm, the convergence and divergence angles of 19.2˚ and 8.31˚, the TKE and its average were 0.2438 m^2^/s^2^ and 0.0305 m^2^/s^2^. then, the TED rate, pressure drop, and reduction in average VD were 0.7151 m^2^/s^3^, 3483.01 Pa, and 2.69 W, respectively). The optimal TKE of 0.0305 m^2^/s^2^ represents the threshold where the effects of turbulence and cavitation are most pronounced. According to Fig 11, at the optimal TKE of 0.0305 m^2^/s^2^, the microbial load showed −2.91 logarithmic reduction. It should also be considered that at higher TKE values, cavitation increases, leading to greater and more effective interaction of microbial cells, resulting in a greater reduction in microbial load, but in this case, energy dissipation also increases and reduces the overall efficiency of the system.

### 3.9. Results of quality assessment of pasteurized milk with a venturi tube reactor

Assessing the quality of milk after treatment is essential to ensure that the process not only eliminates harmful microbes but also preserves the milk’s natural goodness. Key factors like fat, protein, lactose, and SNF reflect the milk’s nutritional value, while the FFp indicates its chemical stability.

Maintaining these properties is vital for preserving the milk’s functional and sensory qualities, such as taste, texture, and overall consumer acceptability. This is especially important for non-thermal methods like the venturi tube reactor, which aim to reduce pathogens effectively without the nutrient loss or flavor changes often associated with traditional heat-based pasteurization.

#### 3.9.1. Milk quality comparison before and after treatment.

While the primary focus of the study is on *E. coli* reduction, it is equally important to evaluate the impact of the non-thermal treatment on the milk’s nutritional and sensory properties. This is because the main advantage of non-thermal pasteurization techniques, like the Venturi tube reactor, is the ability to reduce pathogens without the negative side effects of conventional thermal treatments, which can degrade the quality of the milk.

The table below (Table 4) summarizes the nutritional properties of untreated and treated milk, highlighting the minimal changes observed

**Table 4:** Effect of venturi tube reactor processing on quality properties of Milk.

Property	Untreated Milk	Treated Milk
Fat (%)	3.32	3.16
Protein (%)	3.46	3.38
Lactose (%)	5.01	4.90
Solids-Not-Fat (SNF)	9.21	9.01
Fat-Free portion (FFP) (˚C)	−0.535	−0.524

The minimal changes observed in these key parameters indicate that the non-thermal pasteurization treatment using the venturi reactor does not compromise the milk’s nutritional quality or sensory properties. This is a significant advantage, as it allows for the safe elimination of pathogens like *E. coli* without the negative effects of thermal pasteurization, such as nutrient degradation or off-flavors.

### 3.10. Comparison with previous studies and discussion of strengths and limitations

The overall trends observed in this study are consistent with earlier research on hydrodynamic cavitation in Venturi-based reactors. As reported by Arrojo and Benito (2008) [43], Sliwiński et al. (2020) [44], and Badve and Pandit (2015) [11], throat diameter remains the dominant geometric parameter controlling turbulence intensity, pressure drop, and cavitation-prone structures. The effects of throat length and contraction–expansion angles also follow previously documented CFD findings, where longer throats enhance pressure recovery and larger divergence angles intensify vortex development (Zheng et al., 2021).

Unlike many earlier studies that investigated only individual geometric parameters or relied solely on numerical simulations, the strength of the present work lies in its integrated methodology combining CFD analysis, response-surface optimization, and experimental validation of non-thermal pasteurization performance. This approach not only clarifies how Venturi geometry shapes flow behavior but also demonstrates its practical capability in real processing scenarios, including our separate studies on the non-thermal reduction of *E. coli* and aflatoxin M1 in milk using the same Venturi-based system [23,45].

The main limitation of the present work is that the CFD framework was developed within an early-stage design scope. Accordingly, future studies would benefit from incorporating more advanced simulation models, evaluating the system at pilot and industrial scale conditions, and assessing its performance across a wider range of food matrices. These developments will help extend the applicability of the Venturi-based non-thermal processing system and further strengthen its technological relevance.

## 4. Conclusion

This research aims to design and optimize a venturi tube reactor to create suitable conditions for inducing cavitation phenomena and enhancing TKE during the pasteurization process. This study analyzes the geometric parameters of the venturi tube reactor, specifically the diameter and length of the throat, as well as the angles of convergence and divergence on TKE (both max and ave), the rate of TED, pressure drop, and VD.

The optimization results of the reactor dimensions indicated that the throat diameter had the greatest impact on the dependent variables of this experiment among the independent variables. Simulations of the reactor were conducted using CFD methods to obtain the reactor’s optimal dimensions and calculate the TED and energy dissipation occurring within the venturi tube reactor. According to this simulation and the optimization performed using the RSM, a venturi tube’s throat length should be considered an optimal value, as excessive length does not yield desirable results. Compared to longer ones, a shorter throat length enhances the effects of TKE and cavitation, leading to increased damage to the microbial cell walls through micro-jets and shock waves generated. Additionally, the results indicated that a smaller throat diameter typically results in increased fluid velocity and greater pressure fluctuations than larger diameters, which can enhance TKE and the likelihood of cavitation. This also improves the mixing, mass, and heat transfer efficiency necessary for the pasteurization process. However, it is crucial to note that an excessively small diameter is unsuitable for this process, as it increases TKE and, consequently, the energy dissipation rate. Therefore, it is essential to consider optimal dimensions for constructing this type of reactor. After optimization, the throat diameter and length were determined to be 4.91 mm and 13.15 mm, respectively, along with convergence and divergence angles of 19.2° and 8.31°. The Pearson correlation results of this study also revealed strong and significant statistical relationships between the dependent variables, with parameters such as TKE max, TKE ave, TED, pressure drop and viscous dissipation exhibiting strong positive correlations with one another. Also, Experimental results of milk pasteurization using a venturi tube reactor in laboratory showed that at the optimal TKE of 0.0305 m^2^/s^2^, the microbial load showed −2.91 logarithmic reduction. Additionally, the impact of this pasteurization method on the quality attributes of milk was examined. The strong agreement between CFD-predicted TKE and the experimentally observed microbial reduction confirms the accuracy of the model and validates the reactor’s design concept for practical non-thermal pasteurization applications. The findings demonstrated that while microbial safety was significantly enhanced, the essential sensory and nutritional qualities of the milk were largely maintained. The optimized venturi reactor design can be applied in the food industry for non-thermal pasteurization of milk and other liquid foods, preserving nutritional and sensory quality. This balance between safety and quality highlights the potential of the venturi tube reactor as a viable option for non-thermal pasteurization in the food industry. Future research should aim to validate the reactor’s performance under pilot or industrial scale conditions, optimize its operation for different microbial species, and extend its application to various liquid food matrices.

## Abbreviations

CFD: Computational Fluid Dynamics
RSM: Response Surface Method
TKE: Turbulent Kinetic Energy
CFU: Colony-Forming Units
VD: Viscous Dissipation
TED: Turbulent Energy Dissipation
RANS: Reynolds-Averaged Navier-Stokes
GCI: Grid Convergence Index
SNF: Solid-not-Fat
FFP: Fat-Free Portion
E. coli: Escherichia coli
